# Avian influenza: an osteopathic component to treatment

**DOI:** 10.1186/1750-4732-1-10

**Published:** 2007-07-09

**Authors:** Raymond J Hruby, Keasha N Hoffman

**Affiliations:** 1 Department of Osteopathic Manipulative Medicine, Western University of Health Sciences, College of Osteopathic Medicine of the Pacific, 309 East 2^nd ^Street, Pomona, CA, 91766, USA; 2 Department of Osteopathic Manipulative Medicine, Western University of Health Sciences, College of Osteopathic Medicine of the Pacific, 309 East 2^nd ^Street, Pomona, CA, 91766, USA

## Abstract

Avian influenza is an infection caused by the H5N1 virus. The infection is highly contagious among birds, and only a few known cases of human avian influenza have been documented. However, healthcare experts around the world are concerned that mutation or genetic exchange with more commonly transmitted human influenza viruses could result in a pandemic of avian influenza. Their concern remains in spite of the fact that the first United States vaccine against the H5N1 virus was recently approved. Under these circumstances the fear is that a pandemic of avian influenza could result in the kind of mortality that was seen with the Spanish influenza pandemic of 1918–1919, where the number of deaths was estimated to be as high as 40 million people.

Retrospective data gathered by the American Osteopathic Association shortly after the 1918–1919 influenza pandemic have suggested that osteopathic physicians (DOs), using their distinctive osteopathic manipulative treatment (OMT) methods, observed significantly lower morbidity and mortality among their patients as compared to those treated by allopathic physicians (MDs) with standard medical care available at the time. In light of the limited prevention and treatment options available, it seems logical that a preparedness plan for the treatment of avian influenza should include these OMT procedures, provided by DOs and other healthcare workers capable of being trained to perform these therapeutic interventions. The purpose of this paper is to discuss the characteristics of avian influenza, describe the success of DOs during the 1918–1919 Spanish influenza pandemic, describe the evidence base for the inclusion of OMT as part of the preparedness plan for the treatment of avian influenza, and describe some of the specific OMT procedures that could be utilized as part of the treatment protocol for avian influenza patients.

## Background

Avian influenza, sometimes called "bird flu," is an infection caused by avian influenza virus H5N1, which occurs naturally among birds. Avian influenza is very contagious among birds, but does not usually infect humans; however, cases of human infection with these viruses have occurred since 1997 [1]. At the present time, the virus is known to spread easily only among birds or from birds to humans who have high levels of contact with birds or perhaps bird droppings. There is, however, evidence that the virus could mutate further or exchange genes with a more easily transmitted human influenza virus, setting the stage for larger scale avian influenza infection in humans [2]. Avian influenza has now spread from Asia to Europe, and with the amount of air travel occurring on a daily basis, the thought occurs that human carriers of H5N1 could easily spread the infection around the globe.

The H5N1 virus has a number of characteristics similar to that of the H1N1 virus that caused the influenza pandemic of 1917–1918. The mortality from this pandemic has been variously estimated, but experts agree that as many as 40 million people died worldwide [3]. In data reported by the American Osteopathic Association shortly after the 1918–1919 influenza pandemic, it seemed clear that DOs, using their distinctive OMT methods, observed significantly lower morbidity and mortality among their patients as compared to those treated with commonly used standard medical care available at that time [4]. Given this observation, a logical approach to developing a preparedness plan for the treatment of avian influenza should include these osteopathic procedures. Such procedures can be provided by DOs and other healthcare workers capable of being trained to perform them.

Should avian influenza infection become more widespread among humans, some experts feel that healthcare systems around the world could be thrown into chaos, and that the number of deaths from avian influenza could approach that of the 1918 pandemic [2]. Medications such as oseltamivir (Tamiflu) and zanamivir (Relenza) are at least partially effective against current strains of human influenza viruses. However, there is concern that available quantities of these medications are not enough to protect the world population in the event of a pandemic [5].

The Food and Drug Administration (FDA) recently announced their approval of the first United States vaccine for human use against the avian influenza virus H5N1. In a clinical trial conducted using the vaccine, 103 healthy adults received the recommended dosage, 300 other healthy adults received the vaccine in a dosage lower than that which is recommended, and 48 healthy adults received a placebo injection. Forty-five percent of the individuals who received the recommended dosage developed antibodies at the level that would be expected to reduce their risk of getting avian influenza. Although the remaining individuals developed lower levels of antibodies, they may still have sufficient immunity to reduce the severity of the illness, and the risk of avian influenza-related hospitalization and death [6]. While the success of this vaccine is encouraging, it would seem logical that any and all other methods of prevention and treatment, including OMT procedures, would still be useful in the treatment of avian influenza.

The known data regarding the success of DOs treating influenza were gathered from the 1918 Spanish influenza pandemic and was fist presented by R. Kendric Smith, MD, in a paper in which he described the "osteopathic conquest of disease in which medicine has failed" [7]. Doctor Smith reported that the mortality rate for a total of 110,120 patients with influenza treated by 2445 DOs was 0.25%. Mortality due to influenza in patients receiving traditional medical care, however, was estimated to be 5% to 6%. Patients with pneumonia treated with standard medical care had a mortality rate estimated at 33% overall, and as high as between 68% and 78% in some large cities. Of 6258 patients cared for by osteopathic physicians the death rate due to pneumonia was 10%.

In a paper delivered at the American Osteopathic Association meeting in Chicago in 1919, Riley [8] reported similar low rates of morbidity and mortality from influenza in patients under the care of DOs in large cities such as New York and Chicago. This information suggests that DOs achieved a high success rate in the treatment of patients during the 1918 Spanish influenza pandemic. This may have been due in part to their use of an additional effective therapeutic method – OMT.

These were not controlled studies. The data is retrospective and some conclusions cannot be well drawn from such information. For example, it is not known whether the population of patients treated by DOs was comparable to the population treated by MDs. The remainder of this section describes certain osteopathic research studies from the early 1900s onward that:

• Support the kind of response that was obtained by DOs treating patients afflicted with influenza during the 1918 pandemic.

• Used the same OMT procedures that were used in 1918 to treat patients diagnosed with Spanish influenza.

• Suggest that certain OMT procedures can have a positive stimulating effect on the immune system, possibly allowing the patient to avoid the complications of, and eventually recover from, such illnesses as influenza.

Whiting [9] studied the use of splenic and liver pumps in a group of patients (N = 22), finding that 20 (91%) of the patients had an increase of about 15% in their phagocytic index, the average number of bacteria ingested by each phagocyte after a mixture of blood and bacteria are incubated. Lane [10] experimented with splenic manipulation in rabbits exposed to antigen (sheep erythrocytes). He found that splenic manipulation in rabbits increased antibody levels against these antigens. Castlio and Ferris-Swift [11-13] described changes induced by splenic manipulation in asymptomatic subjects. They used a technique that consisted of applying alternating compressions to the spleen for 1.5 to 5 minutes at a rate of 21 compressions per minute. They reported an increase in leukocyte count in 80% of the cases studied, with a decrease in erythrocyte count in 75% of the cases. They also found an increase in opsonic index in more than 80% of the cases and an increase in serum bacteriolytic power in 68% of the cases. They concluded that the increased leukocyte count was the result of "contraction of the spleen and expulsion of its contained leukocytes," and that the decreased erythrocyte count was due to increased destruction of red blood cells by the spleen.

Measel [14] studied the effect of lymphatic pumps on the immune response of male medical students who were vaccinated with pneumococcal polysaccharide (Pneumovax). He used bacterial agglutination and passive agglutination tests to assess immune response to pneumococcal polysaccharide as an antigen. At 14 days, students who received lymphatic pump treatment had a statistically greater immune response than the control group, which received no postimmunization treatment. The conclusion was that lymphatic pump treatments had a positive effect on the B-cell and T-cell components of the human immune system as measured in peripheral blood. In a 1986 study, Measel and Kafity [15] used a lymphatic pump technique to demonstrate an increase of white blood cell count in peripheral blood, from 7460 erythrocytes per dL to 9810 per dL. The B-cell component increased from 5.07% to 9.25%, while the T-cell component rose from 73.2% to 80.9%. Jackson and Steele et. al. [16,17] explored the effect of lymphatic and splenic pump procedures on the antibody response to a hepatitis B vaccine. The experimental subjects received lymphatic and splenic pump treatments three times per week for two weeks after each vaccination. Subjects were given the vaccinations at 0, 5, and 25 weeks. The control group received the vaccinations but no OMT. Fifty percent of the subjects in the treatment group achieved protective antibody titers by week 13 compared with the same result in only 16% of the control subjects. The results were presented as further evidence that the lymphatic and splenic pumps enhance immune response.

Mesina et. al. [18] and Hampton et. al. [19] reported on male subjects who received lymphatic pump treatments consisting of pectoral traction and splenic pump. A control group did not receive lymphatic pump treatment. Blood samples were collected at 15, 30, 60, 120, and 240 minutes after treatment. All subjects in the treatment group showed significant basophilia, a condition that may play a significant role in initial immune response.

Sleszynski and Kelso [20] conducted a trial of the thoracic lymphatic pump in patients who had undergone low-risk cholecystectomy. Half of the subjects received treatment with the thoracic lymphatic pump, while the other half received incentive spirometry. Atelectasis occurred in two patients in each group. However, patients treated with the thoracic lymphatic pump had earlier recovery and quicker return to preoperative values of forced vital capacity than did those treated with incentive spirometry.

Some animal studies have also supported the effectiveness of lymphatic pump procedures. A study by Dery et. al., [21] using normal laboratory rats, suggested that lymphatic pumps can increase the distribution rate of lymph. Another study by these same authors suggested that in rats mechanical pressure (similar to thoracic pumps) applied to regions of the body distant from the location of lymph formation can enhance lymph uptake [22].

A study by Knott et. al. [23] suggested that in dogs both thoracic and abdominal pumps caused increases in real-time measurements of lymph flow through the thoracic duct, from 1.20± 0.41 to 3.45± 1.61 mL/minute. These effects occurred without changes in other hemodynamic measurements, including mean arterial pressure, heart rate, and cardiac output.

Washington et.al. [24] compared an experimental group of hospitalized patients with an admitting diagnosis of pneumonia to a control group without an admitting diagnosis of pneumonia. They noted that those patients who had pneumonia had a high predictability of presenting with Chapman reflex points related to the lungs. Chapman reflexes are defined as follows: "A system of reflex points that present as predictable anterior and posterior fascial tissue texture abnormalities (plaque-like changes or stringiness of the involved tissues) assumed to be reflections of visceral dysfunction or pathology" [25]. These reflex points were discovered by Frank Chapman, DO, and later described in a book by Charles Owens, DO [26]. Chapman reflex points may be used diagnostically as well as therapeutically. In this paper, we shall consider the treatment of certain Chapman reflex points as potentially useful for patients with influenza. The specific points are described later in this paper.

DOs need to be well prepared to treat patients who may develop avian influenza. To be prepared one should:

▪ Understand how to recognize the clinical characteristics of the avian influenza infection.

▪ Have a preparedness plan for the treatment of patients with avian influenza infection.

▪ Be able to perform the OMT procedures that could have a beneficial effect on the patient with avian influenza.

### What is Avian Influenza?

#### Clinical Characteristics

As mentioned above, avian influenza, or "bird flu," is an infection caused by avian influenza viruses, which occur naturally among birds. The precise length of the incubation period for bird influenza in humans isn't known, but the illness commonly develops within one to five days of exposure to the virus. On occasion, the only sign of the disease is a relatively mild conjunctivitis, but more frequently, signs and symptoms of bird flu resemble those of conventional influenza, including:

▪ Cough

▪ Fever

▪ Sore throat

▪ Myalgias

Patients infected with the H5N1 form of the virus (the most virulent type) may develop life-threatening complications, such as viral pneumonia and acute respiratory distress, the most common cause of death from bird flu [27].

#### Treatment

The primary treatment option is the influenza drug oseltamivir (Tamiflu). This is a neuraminidase inhibitor whose mechanism of action is prevention of the virus from escaping its host cell. It is not clear how effective Tamiflu will prove to be against the H5N1 virus. Reports from Southeast Asia indicate that resistance to H5N1 is quickly developing. Another antiviral influenza drug, zanamivir (Relenza), is an alternative form of pharmaceutical treatment. However, both of these drugs must be taken within 48 hours of the appearance of symptoms, which may prove difficult in the event of a pandemic and the fact that these agents are currently in short supply [27].

Based on the results (discussed above) of the use of OMT during the 1918 Spanish influenza pandemic, we propose that OMT be included as a part of the overall treatment plan for patients with influenza. In particular, we propose that the use of OMT in such a treatment plan for avian influenza could be effective in preventing the kind of overall morbidity and mortality that was associated with the pandemic of 1918. The mechanisms by which the OMT procedures proposed here would be of benefit in the treatment of avian influenza could be classified in the following categories: 1) those procedures known to enhance the patient's immune response, providing the patient with a means to further avoid complications and promote recovery, and 2) those procedures that would improve body mechanics by way of reducing tissue hypertonicity and joint hypomobility. These latter procedures would provide such biomechanical improvements as optimal rib cage and thoracoabdominal diaphragm motion, which in turn would result in improved respiratory mechanics, and improved arterial, venous and lymphatic circulation throughout the body. This result, coupled with the patient's enhanced immune system response, would provide a more optimum internal environment for the patient to recover from an illness such as avian influenza.

#### Prevention

As mentioned above, the FDA recently announced their approval of the first United States vaccine for human use against the avian influenza virus H5N1. While the success of this vaccine is encouraging, it would seem logical that in addition to the use of the vaccine, prevention will require the use of other known useful precautionary measures. Immediate measures include: [28]

1. Isolation of clinical cases of moderate-to-severe respiratory disease and other patients under investigation in respiratory isolation rooms or single rooms.

2. Identification and voluntary home quarantine of asymptomatic close contacts and daily monitoring for symptom onset.

3. Administration of antiviral drugs for the treatment of cases and, if domestic supplies permit, for the targeted prophylaxis of close contacts.

4. Strict infection control and the use of personal protective equipment in health care facilities caring for cases during the delivery of health care.

5. Intensive promotion of hand and cough hygiene.

6. Domestic cleaning, using household cleaning products, to reduce transmission via fomites (infectious respiratory secretions on surfaces).

A comprehensive pandemic influenza plan has been published by the United States Department of Health and Human Services [29].

## Conclusion

OMT proved to be a critical factor in the success of osteopathic physicians treating influenza patients during the pandemic of 1918 [7]. Subsequent research has shown that certain OMT procedures can stimulate the immune system, and others can improve arterial, venous and lymphatic circulation by way of improving such things as rib cage biomechanics and thoracoabdominal diaphragm motion. Accomplishment of these treatment goals may provide the mechanism for avoidance of complications and an increased rate of recovery from such illnesses as influenza.

DOs must be prepared to provide these beneficial treatments to patients afflicted with avian influenza. Colleges of osteopathic medicine can train their students to provide these treatments as well. Osteopathic interns and residents can also be prepared to provide the appropriate OMT procedures. As a result, there would be a critical mass of osteopathic physicians and osteopathic physicians-in-training available to provide this important and proven osteopathic component to the care of those who become infected with the avian influenza virus. Interested allopathic physicians and physicians-in-training, and other qualified healthcare workers could also be instructed in how to perform these OMT procedures. Given the success rate of osteopathic physicians during the 1918 influenza pandemic, the use of OMT in the treatment of avian influenza infection could help to significantly reduce morbidity and mortality.

### OMT procedures useful in the treatment of influenza

The following section describes some of the OMT procedures that could be useful in the treatment of patients with avian influenza infection. Included here are descriptions of the specific procedures discussed earlier in the literature review. These include the thoracic, hepatic, splenic, abdominal and pedal lymphatic pump procedures, and rib raising procedures. Also included are other OMT procedures that, although not thoroughly researched, have been clinically observed to provide similar effects. These procedures include soft tissue procedures, pectoral traction, mandibular drainage, frontal and maxillary lifts, and diaphragm doming. Specific Chapman reflexes, observed to be useful in treatment of respiratory ailments such as influenza are shown. Finally, muscle energy techniques that can help to improve rib cage biomechanics, are described. These OMT procedures are not presented as a specific treatment protocol, but rather as a listing of OMT procedures as a resource for use in an overall treatment plan for a given patient.

### Specific evidence-based OMT procedures

#### Thoracic lymphatic pump technique

##### Classical thoracic pump technique

**Figure 1:**
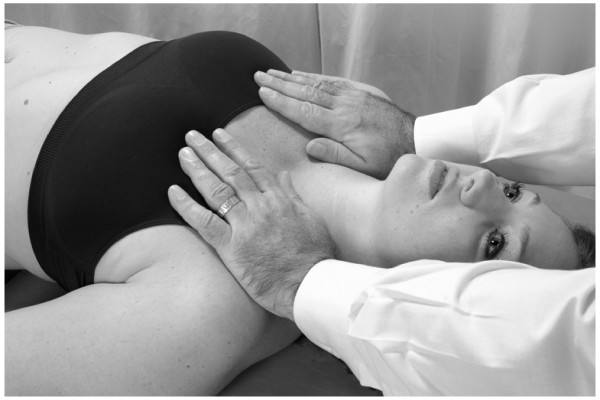
Classical thoracic pump technique.

• The physician stands at the head of the supine patient, placing both hands on the thoracic wall with the thenar eminence of each hand just distal to the respective clavicle, and his/her fingers spreading out over the chest wall (Fig. 1).

• In the female patient it is important not to apply heavy pressure to the breast. However, gentle pressure can assist in lymph drainage of congested breasts.

• The physician induces a rhythmic pumping action by alternating pressure and release with the hands. The motion is generated through a slight extension/flexion of elbows, with forearm, wrist and hand acting as a fixed lever. The oscillatory force of the motion should come from the physician's whole body.

• The rate of the pumping should be approximately 110–120 times/minute.

• The patient continues to breathe normally during this treatment.

• The physician continues until a palpatory sense of increased soft tissue compliance, decreased tissue congestion, is attained.

##### Thoracic pump variation with activation

A modification of the thoracic pump technique is to induce the pumping action while the patient is inhaling and then exhaling.

**Figure 2:**
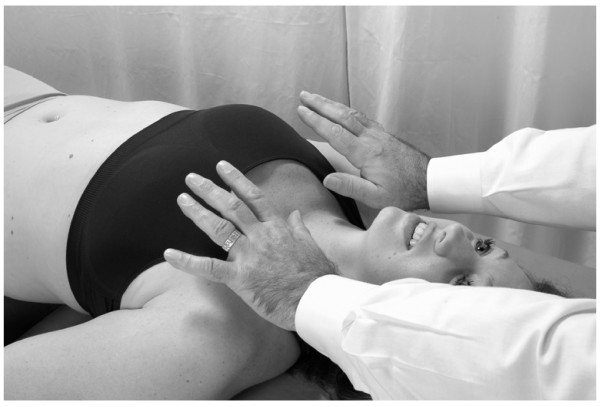
Abrupt hand release for thoracic lymphatic pump technique with activation.

• When the patient reaches full exhalation, the physician maintains a firm, steady pressure on the thoracic wall. This cycle of inspiration and expiration is repeated typically 4–5 times, but varies according to the patient's needs and response to treatment.

• With each subsequent breath, the physician maintains the compressive force at the end point of exhalation.

• On the fourth or fifth inhalation, the patient is told to hold his/her breath in exhalation.

• When the patient feels the need to inspire, the physician instructs the patient to take a sudden, deep breath in. At this point, the physician abruptly releases the pressure on the thoracic wall (Fig. 2).

#### Hepatic pump technique

##### Hepatic pump: supine position

**Figure 3:**
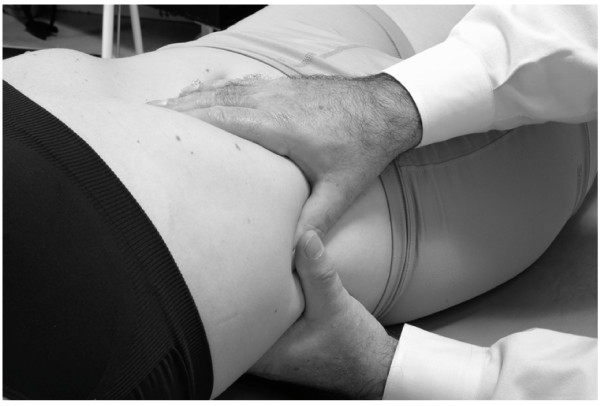
Hand position for hepatic pump technique in the supine position.

• The physician stands on the right side of the supine patient, facing the head.

• The physician passes the left hand underneath the lower ribs and the right hand on the abdominal wall immediately below the costal margin.

• The patient takes in a deep breath while the physician identifies the inferior border of the liver with the tips of the fingers of the right hand.

• As the exhalation occurs, the fingers penetrate the abdominal soft tissue over the liver and underneath the costal margin.

• The patient takes another deep breath. This time during exhalation, a vibratory force is applied through the right hand on the liver (Fig. 3).

• This process is repeated, each time penetrating a little deeper into the subcostal area, gaining more contact with the liver. The technique is continued until liver congestion (and related tenderness) is reduced as much as possible.

##### Hepatic pump: lateral recumbent position

**Figure 4:**
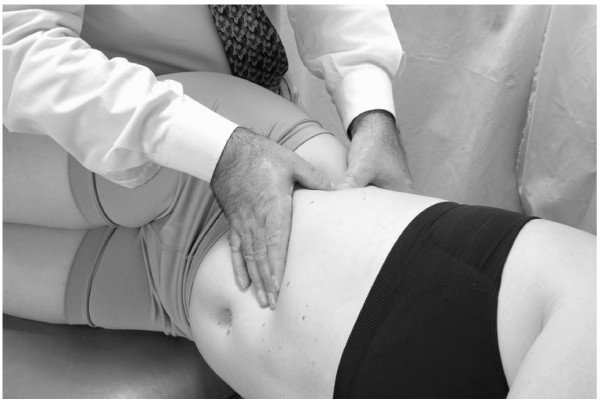
Hand position for hepatic pump technique in the lateral recumbent position.

**Figure 5:**
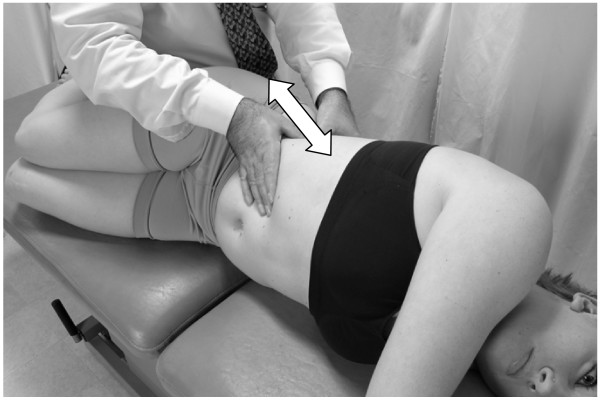
Hand motion for lateral recumbent hepatic pump technique.

• The patient is positioned in a left lateral recumbent position with the hips and knees flexed to stabilize the body.

• The physician is seated on the table (Fig. 4).

• The physician places both hands on the lower thoracic cage with the right hand anteriorly, the left hand posteriorly, and the thumbs meeting in the axillary line.

• The patient takes a deep breath. As the patient exhales, the physician applies a vibratory motion with both hands to induce the pumping action to the liver (Fig. 5).

#### Splenic pump

##### Splenic pump: supine position

**Figure 6:**
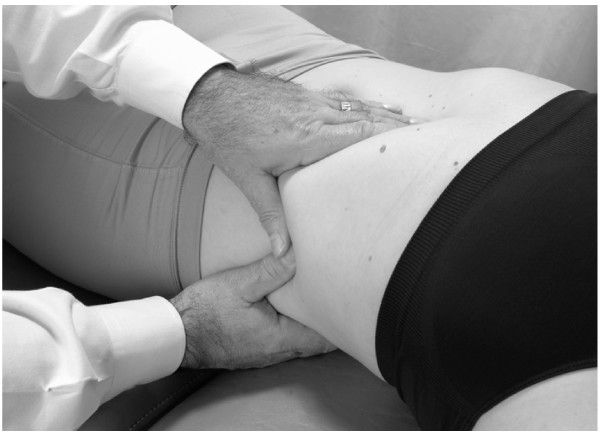
Hand position for supine splenic pump technique.

**Figure 7:**
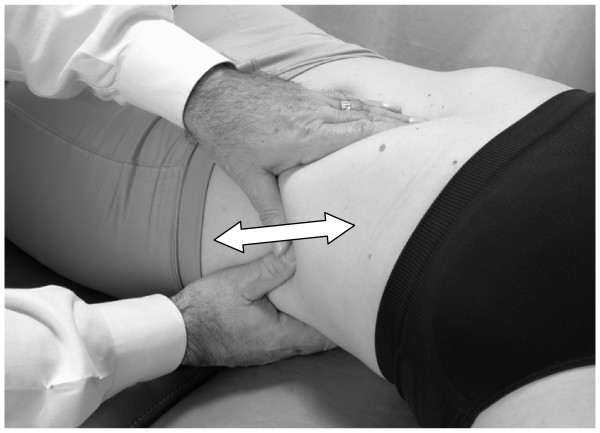
Hand movement for supine splenic pump technique.

• This technique is identical to the supine liver pump, except that all of the directions are reversed to reflect the fact that the spleen is located on the left side of the body (Figs. 6 and 7).

##### Splenic Pump: lateral recumbent position

**Figure 8:**
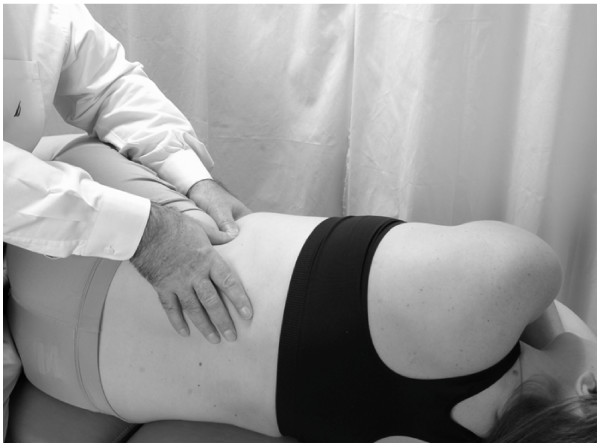
Hand position for lateral recumbent splenic pump technique.

**Figure 9:**
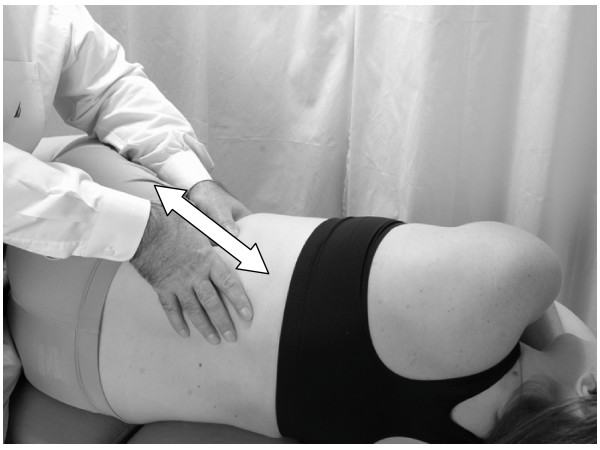
Hand movement for lateral recumbent splenic pump technique.

This technique is identical to the lateral recumbent liver pump, except that all of the directions are reversed to reflect the fact that the spleen is located on the left side of the body (Figs. 8 and 9).

#### Abdominal lymphatic pump

**Figure 10:**
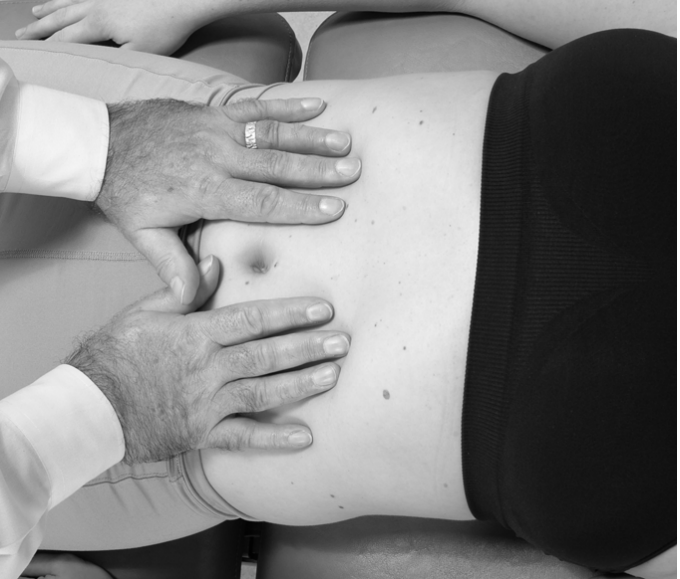
Abdominal pump technique.

• The patient is supine with the physician standing, or kneeling on the table at the patient's side.

• The physician places both palms on the patient's abdomen with fingers pointing to the patient's head, thumbs side by side (Fig. 10).

• Both arms should be extended and elbows locked.

• The physician then pumps in a rhythmic manner. The pumping motion is similar to the pedal or thoracic pump. This rate should be approximately 20–30 times/minute, but varies according to the patient's needs and response to treatment.

#### Pedal lymphatic pump

**Figure 11:**
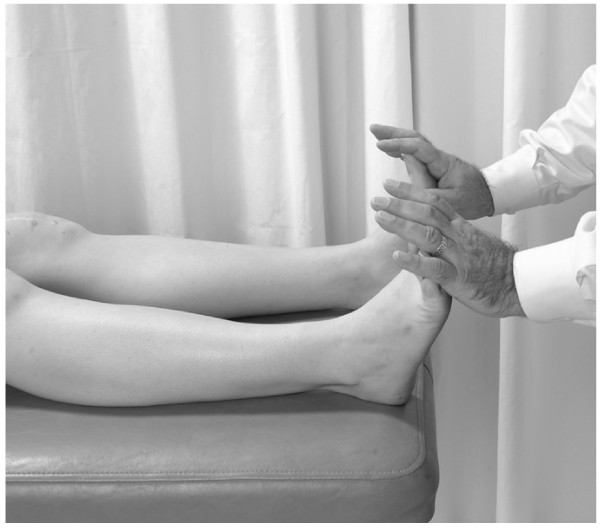
Starting position for pedal pump technique.

• The physician stands at the feet of the supine or prone patient.

• The physician dorsiflexes the patient's feet (Fig. 11).

• The physician introduces a force that dorsiflexes the feet (past neutral). The umbilicus may be used as a landmark to appreciate a wave of motion.

• As the rebound wave returns to the feet, a dorsiflexion force is reapplied, thereby creating an oscillatory pump.

#### Rib raising procedures

##### Method 1

Position: The patient is seated facing the physician. The physician stands in front of the patient.

**Figure 12:**
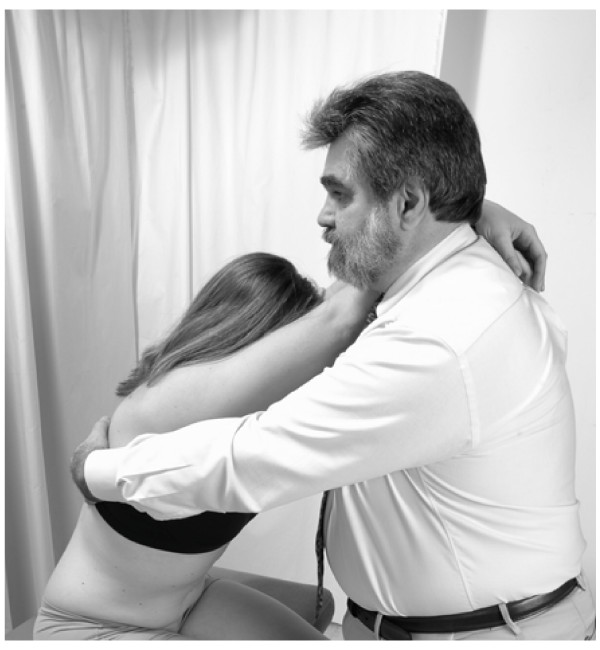
Rib raising technique – method 1.

1. The patient crosses his/her arms and places both arms over the physician's shoulder.

2. The patient's head rests on his/her arms.

3. The physician reaches behind the patient with both arms to contact the rib angles medially with the finger pads as a fulcrum for extension of the patient's spine.

4. The physician simultaneously applies an anterior-lateral traction on the contacted rib angles and extends the patient's spine by shifting the physician's center of gravity posteriorly and pulling the patient towards the physician to stretch the intercostal spaces and engage the restrictive barrier, then returns the patient towards neutral (Fig. 12).

5. The physician repeatedly engages this restrictive barrier in a rhythmic fashion until there is increased range of motion towards the physiological barrier.

6. Retest.

##### Method 2

Position: The patient is supine. The physician sits facing the table on the side of rib restriction.

**Figure 13:**
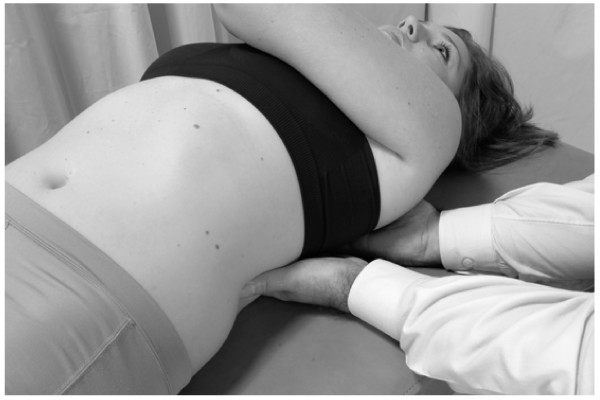
Rib raising technique – method 2.

1. The physician reaches underneath the patient with both arms to contact the rib angles medially with the finger pads. The contact between the physician's metacarpophalangeal joints and the table will serve as a fulcrum.

2. The physician applies an anterolateral traction on the contacted rib angles by flexing the wrists to mobilize costotransverse and costovertebral joints and engage the restrictive barrier for, then returns the patient's ribs towards neutral (Fig. 13).

3. The physician repeatedly engages this restrictive barrier in a rhythmic fashion until there is increased range of motion towards the physiological barrier.

4. Retest.

#### Other useful OMT procedures

##### Pectoral traction

**Figure 14:**
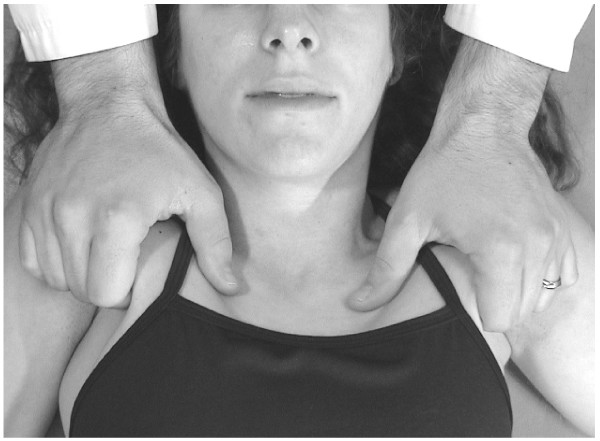
Pectoral traction technique.

• The physician stands at the head of the supine patient.

• The physician gently grasps the inferior border of the pectoralis muscles of each anterior axilla, taking care not to gouge the patient with the fingertips (Fig. 14).

• With arms fully extended, the physician produces a bilateral superior traction by leaning back and using the entire body.

While maintaining traction, the physician instructs the patient to breathe deeply. The combination of traction and respiratory motion releases the upper anterior thoracic muscle tension.

##### Mandibular drainage (Galbreath) technique

**Figure 15:**
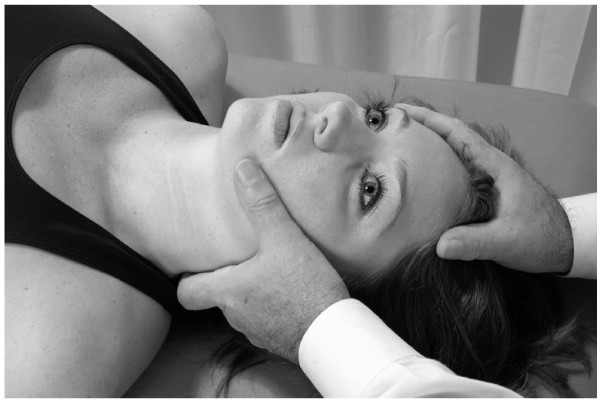
Mandibular drainage (Galbreath) technique.

• The patient is supine or seated.

• The physician uses one hand to stabilize the patient's head.

• With his/her other hand, the physician contacts the patient's mandible on the side to be treated.

• The physician exerts a gentle inferior and medial traction on the patient's mandible for 3–5 seconds and then allows the mandible to return to the neutral position (Fig. 15).

• This motion is repeated in a rhythmic fashion until soft tissue tension is released. The technique takes about 30–60 to apply. It may be repeated if necessary in the same treatment session.

##### Sinus drainage procedures

**Figure 16:**
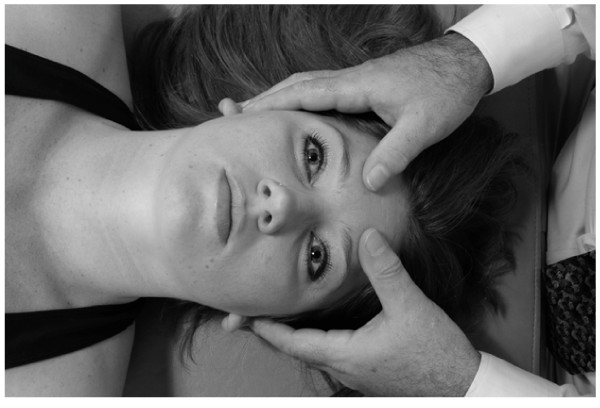
Frontal sinus drainage technique.

**Figure 17:**
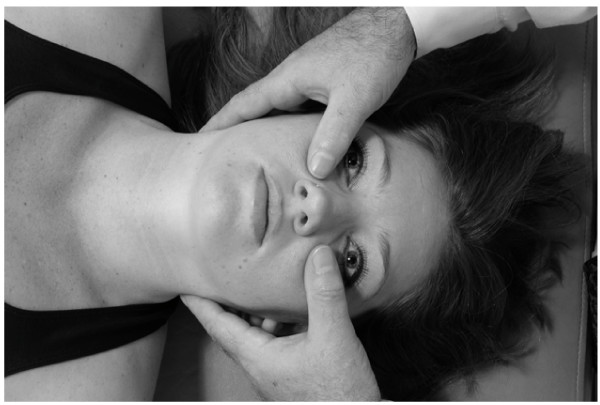
Maxillary sinus drainage technique.

• The patient is supine.

• The physician is seated at the head of the treatment table.

• The physician places the pads of his/her thumbs on the patient's frontal bone, contacting the area over the frontal sinuses.

• The physician then applies gentle pressure over the frontal sinuses with the pads of the thumbs, and then slowly glides the thumbs laterally. The thumbs are then placed back in the starting position over the frontal sinuses (Fig. 16).

• This motion is repeated over a 30 – 60 second period.

• This technique can be performed in a similar fashion on the maxillary sinuses (Fig. 17).

##### Frontal lift

**Figure 18:**
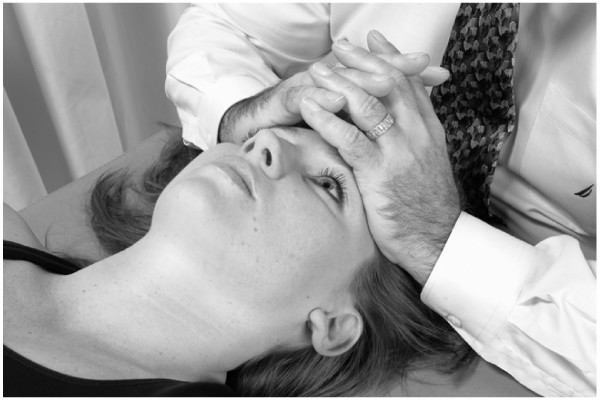
Frontal lift technique.

• The patient is supine.

• The physician sits at the head of the table.

• The physician rests his/her elbows on the table.

• The physician places his/her hypothenar eminences on the lateral angles of the frontal bone and interlaces the fingers over, but not in contact with, the forehead (Fig. 18).

• The physician applies a small amount of medial pressure bilaterally with the hypothenar eminences, thus allowing the hypothenar eminences of both hands to

• grip the lateral angles of the frontal bone.

• The physician then gently lifts the frontal bone anteriorly (toward the ceiling) until the limit of frontal bone motion and associated soft tissue tensions are reached.

• The physician maintains this position until the tissue tension.

• The physician then gently releases his/her hands.

##### Maxillary lift

**Figure 19:**
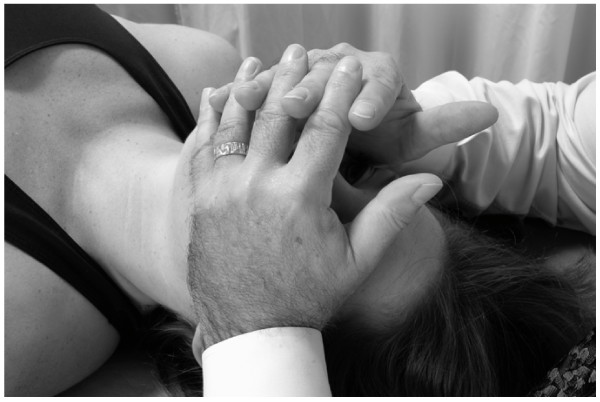
Maxillary lift technique.

The maxillary lift technique is performed in the same manner as the frontal lift technique described above. The physician's hands, however, are repositioned so that the hypothenar eminences are in contact with the lateral aspects of the maxillary bones (Fig. 19).

#### Doming of diaphragm

##### Supine doming of the diaphragm

**Figure 20:**
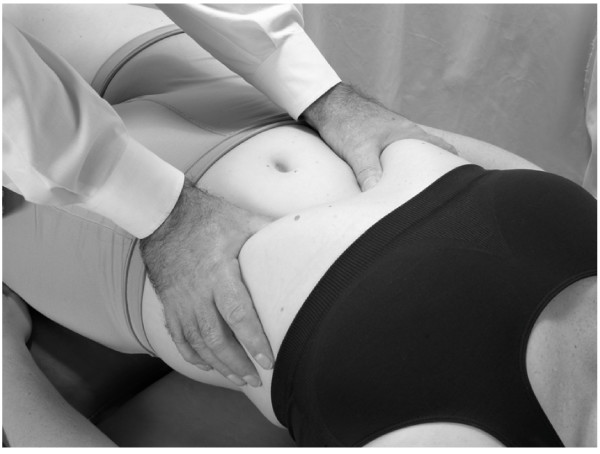
Supine doming of the diaphragm.

• The patient is lying supine with the physician standing at the patient's side.

• The physician places the fingers of both hands on the outer aspect of the inferior border of the ribs with thumbs pointed toward each other medially and positioned directly inferior to the xiphoid process of the sternum (Fig. 20).

• The patient inhales deeply and then exhales fully. As the patient exhales, the physician gently presses the thumbs posterior toward the table, palpating the motion of the diaphragm.

• The physician holds this end point as the patient inhales again. The thumbs should sense resistance to the respiration.

• As the patient exhales again, the physician presses in further posteriorly toward the table, continuing to monitor the superior movement of the diaphragm.

This process is continued through several respiratory cycles until the diaphragm domes easily at the end of exhalation.

#### Chapman reflexes

**Figure 21:**
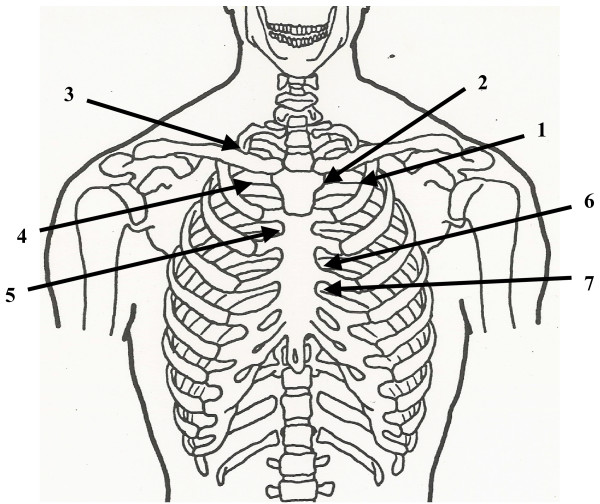
Anterior Chapman points suitable for use in treating patients with influenza: 1. Sinusitis. 2. Nasal. 3. Otitis media. 4. Pharyngitis. 5. Bronchitis. 6. Upper lung. 7. Lower lung.

**Figure 22:**
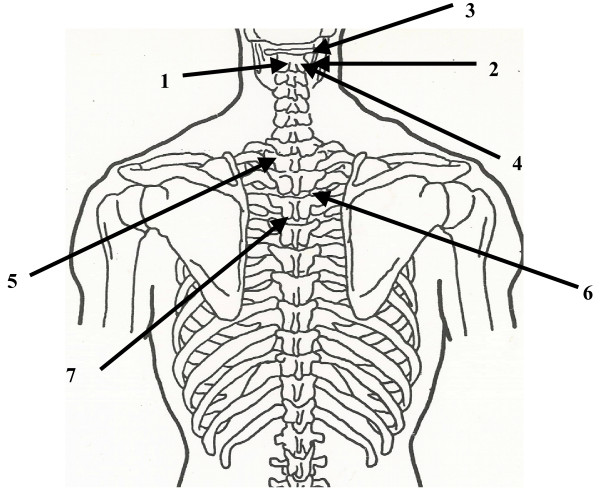
Posterior Chapman points suitable for use in treating patients with influenza: 1. Sinusitis. 2. Nasal. 3. Otitis media. 4. Bronchitis. 5. Upper lung. 6. Lower lung.

Chapman reflexes were developed by Frank Chapman, DO, and described by Charles Owens, DO [26]. This is a system of reflex points that present as anterior and posterior fascial tissue texture abnormalities and are assumed to be reflections of visceral dysfunction or pathology. The treatment of these points consists of applying light pressure to the point with the pad of the index finger or thumb and then providing a circular motion to the point until it is dissipated. Certain Chapman reflex points may be of value in treating patients with influenza (Figs. 21 and 22):

Sinusitis: An anterior point located approximately 3.5 inches lateral to the sternum and on the superior edge of the second rib; a posterior point located midway between the spinous process and the tip of the transverse process of C2.

Nasal: An anterior point located on the first rib at the junction of the rib with the sternocostal cartilage; a posterior point located on the tip of the transverse process of C2.

Otitis Media: An anterior point on the superior surface of the clavicle just lateral to where it crosses the first rib; a posterior point on the superior edge of the posterior aspect of the tip of the transverse process of C1.

Pharyngitis: An anterior point located on the anterior surface of the first rib; a posterior point midway between the spinous process and the tip of the transverse process of C2, on the posterior aspect of the transverse process.

Bronchitis: An anterior point located in the second intercostal space near the edge of the sternum; a posterior point located on the posterior surface of the transverse process of T2, midway between the spinous process and the tip of the transverse process.

Upper Lung: An anterior point located in the third intercostal space near the edge of the sternum; a posterior point located in the intertransverse space, midway between the spinous processes and the tips of the transverse processes of T3 and T4.

Lower Lung: An anterior point located in the fourth intercostal space near the edge of the sternum; a posterior point located in the intertransverse space, midway between the spinous processes and the tips of the transverse processes of T4 and T5.

Muscle energy procedures for the rib cage

Muscle energy technique (MET) is defined as: "A system of diagnosis and treatment in which the patient voluntarily moves the body as specifically directed by the osteopathic practitioner. This directed patient action is from a precisely controlled position against a defined resistance by the osteopathic practitioner"[25].

#### MET for rib inhalation somatic dysfunction

##### Rib 1

Diagnosis: Rib 1 on the left – inhalation somatic dysfunction

Restriction: Rib 1 on the left demonstrates reduced ability to move into the exhalation position

Position: The physician stands at the head of the supine patient

**Figure 23:**
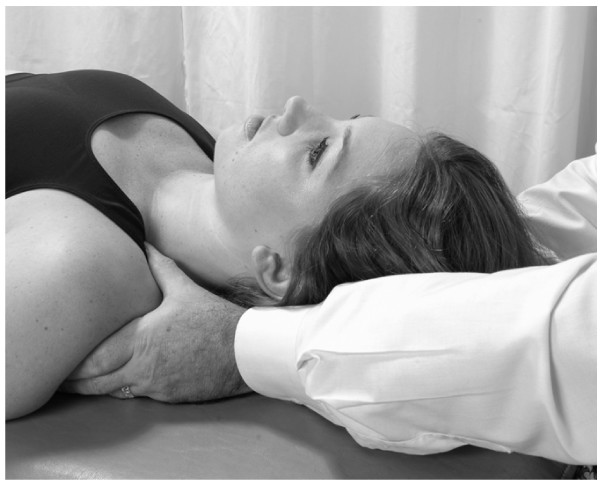
Inhalation restriction of left first rib. Hand position for muscle energy technique.

1. The physician monitors the head of the dysfunctional rib in the supraclavicular fossa with his/her left thumb.

2. The head is flexed using the right hand until motion is felt at rib one (Fig. 23).

3. The patient inhales and exhales deeply. As the patient exhales, rib 1 is moved inferiorly into the restrictive barrier. The patient holds his/her breath in exhalation for 3–5 seconds.

4. While holding his/her breath in exhalation, the patient should then push his/her head backwards against the physician's unyielding counterforce. This isometric contraction should last for the 3–5 seconds while the patient is holding his/her breath.

5. When the patient inhales, the physician resists the natural tendency of the rib to move superiorly with inhalation.

6. Steps 3–5 are repeated 3–5 times with the physician reengaging a new restrictive barrier with each repetition. Following the final repetition, a final stretch is performed further into the restrictive barrier.

7. Retest.

##### Ribs 2–5

Diagnosis: Rib 2 on the left – inhalation somatic dysfunction

Restriction: Rib 2 on the left demonstrates reduced ability to move into the exhalation position

Position: The physician stands at the head of the supine patient

**Figure 24:**
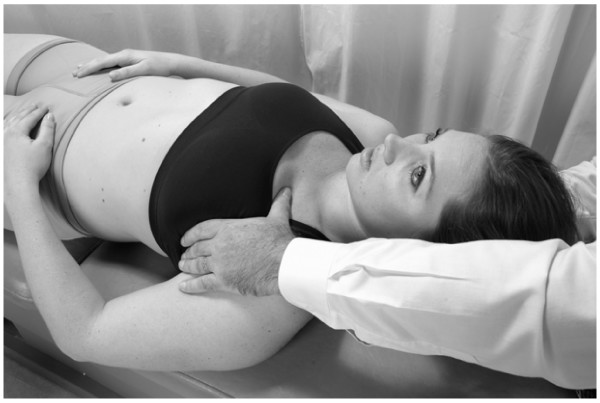
Inhalation restriction of left secondt rib. Hand position for muscle energy technique.

1. The web space formed by the thumb and index finger of the physician's left hand should be placed in the first intercostal space above the dysfunctional rib (rib 2) on the anterosuperior surface of the rib.

2. Using his/her right hand, the physician flexes the patient's head until motion is felt at rib 2 (Fig. 24).

3. The patient inhales and exhales deeply. As the patient exhales, the physician moves rib 2 inferiorly into the restrictive barrier with his/her left hand. The patient holds his/her breath in exhalation for 3–5 seconds.

4. As the patient holds his/her breath in exhalation, he/she performs an isometric contraction by pushing his/her head backward against the physician's unyielding counterforce.

5. As the patient inhales, the physician resists the natural tendency of the rib to move superiorly during inhalation.

6. Steps 3–5 are repeated 3–5 times with the physician reengaging a new restrictive barrier with each repetition. Following the final repetition, a final stretch is performed further into the restrictive barrier.

7. Retest.

##### Ribs 6–10

Diagnosis: Rib 8 on the left – inhalation somatic dysfunction

Restriction: Rib 8 on the left demonstrates reduced ability to move into the exhalation position

Position: The physician stands on the side of the dysfunctional rib near the head of the supine patient

**Figure 25:**
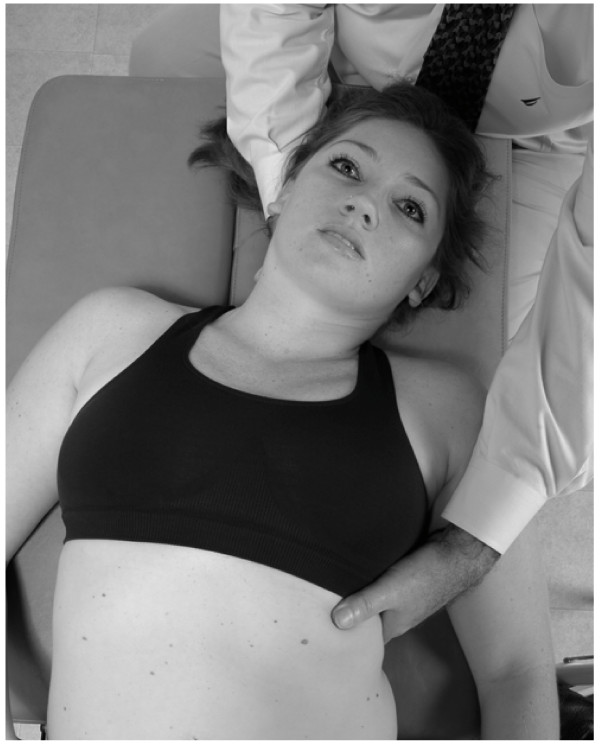
Inhalation restriction of left eigth rib. Superior view of hand position for muscle energy technique.

1. The physician places the web space between the index finger and thumb of the left hand in the 7^th ^intercostal space, above rib 8.

2. The physician flexes and sidebends the patient's head towards the side of the dysfunctional rib. The patient reaches toward his/her left foot with the left hand.

3. The patient inhales and exhales deeply, and during exhalation the patient also reaches toward his/her left foot while the physician moves the rib inferiorly with his/her left hand to engage a new restrictive barrier (Fig. 25).

4. The patient holds his/her breath in exhalation for 3–5 seconds while pushing his/her head backwards against the physician's unyielding counterforce.

5. Steps 3–5 are repeated 3–5 times with the physician reengaging a new restrictive barrier with each repetition. Following the final repetition, a final stretch is performed further into the restrictive barrier.

6. Retest.

##### Ribs 11–12

Diagnosis: Rib 12 on the right – inhalation somatic dysfunction

Restriction: Rib 12 on the right demonstrates reduced ability to move into the exhalation position

Position: The physician stands on the left side of the prone patient

**Figure 26:**
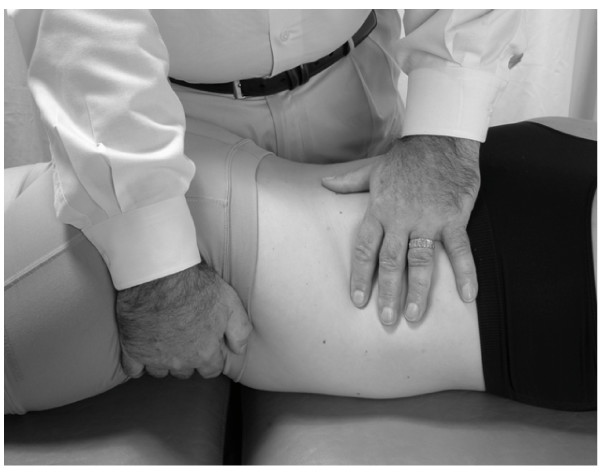
Muscle energy technique for inhalation restriction of right 12^th ^rib.

1. The thenar eminence of the physician's left hand is placed just above the superior border and medial to the rib angle and exerts an inferolateral pressure on the right 12^th ^rib.

2. The physician's right hand grasps the ipsilateral ASIS and lifts the patient's innominate superiorly to engage the restrictive barrier.

3. The patient is instructed to pull his/her right hip toward the table against the physician's unyielding counterforce and this isometric contraction is held for 3–5 seconds (Fig. 26).

4. The patient then relaxes, and the physician releases his/her counterforce until the tissues relax for 2–3 seconds.

5. Steps 3 and 4 are repeated 3–5 times with the physician reengaging a new restrictive barrier with each repetition. Following the final repetition, a final stretch is performed further into the restrictive barrier.

6. Retest.

#### MET for rib exhalation somatic dysfunction

##### Rib 1

Diagnosis: Rib 1 on the left – exhalation somatic dysfunction

Restriction: Rib 1 on the left demonstrates reduced ability to move into the inhalation position

Position: The physician stands on the right side of the supine patient

**Figure 27:**
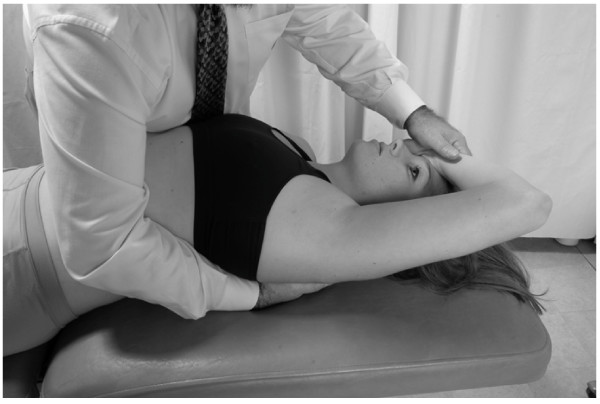
Muscle energy technique for exhalation restriction of left first rib.

1. The physician reaches with the right hand under the patient to grasp the rib angle of the dysfunctional first rib and applies traction in an inferolateral direction.

2. The physician places the dorsum of the patient's left wrist on the patient's forehead and the physician places his/her left hand over the patient's wrist. The patient's head is lying straight ahead (Fig. 27).

3. The patient inhales while the physician moves the rib inferiorly to engage the restrictive barrier.

4. The patient holds in his/her breath for 3–5 seconds while contracting the anterior/middle scalenes to his/her head toward the ceiling against the physician's unyielding counterforce.

5. Steps 3 and 4 are repeated 3–5 times with the physician re-engaging a new restrictive barrier after each repetition. Following the final repetition, a final stretch is performed further into the restrictive barrier.

6. Retest.

##### Rib 2

Diagnosis: Rib 2 on the left – exhalation somatic dysfunction

Restriction: Rib 2 on the left demonstrates reduced ability to move into the inhalation position

Position: The physician stands on the right side of the supine patient

1. The physician reaches with the right hand under the patient to grasp the rib angle of the dysfunctional 2^nd ^rib and applies traction in an inferolateral direction.

2. The physician places the dorsum of the patient's left wrist on the patient's forehead and then places his/her left hand over the patient's wrist. This position is similar to that described for rib 1 above, with the exception that the patient's head is rotated 30° to the right (contralateral direction) for better contraction of the posterior scalene muscle.

3. The patient inhales while the physician moves the rib inferiorly to engage the restrictive barrier.

4. The patient holds in his/her breath for 3–5 seconds while contracting the posterior scalene to lift his/her head towards the ceiling against the physician's unyielding counterforce.

5. Steps 3 and 4 are repeated 3–5 times with the physician reengaging a new restrictive barrier after each repetition. Following the final repetition, a final stretch is performed further into the restrictive barrier.

6. Retest.

##### Ribs 3–5

Diagnosis: Rib 4 on the left – exhalation somatic dysfunction

Restriction: Rib 4 on the left demonstrates reduced ability to move into the inhalation position

Position: The physician stands on the right side of the supine patient

**Figure 28:**
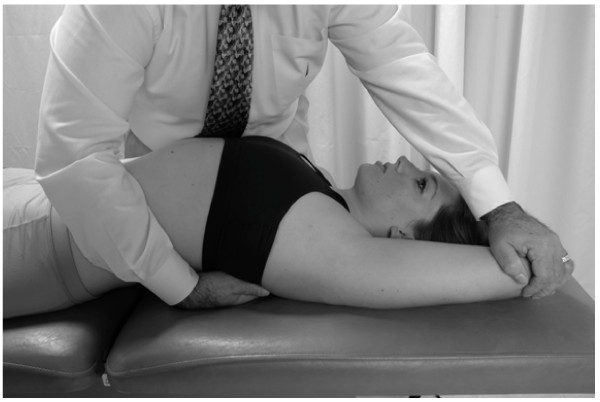
Muscle energy technique for exhalation restriction of left fourth rib.

1. The physician reaches with the right hand under the patient to grasp the rib angle of the dysfunctional 4^th ^rib and applies traction in an inferolateral direction.

2. The physician flexes the patient's left elbow to 90° and lifts the arm, placing the hand above the head of the patient with the elbow adjacent to the patient's head and the arm resting on the table. The physician places his/her right hand on the patient's elbow. The patient's head is lying straight ahead.

3. The patient inhales while the physician moves the rib inferiorly into the restrictive barrier.

4. The patient holds in his/her breath for 3–5 seconds while contracting the pectoralis minor to bring his/her left elbow toward the right ASIS against the physician's unyielding counterforce (Fig. 28).

5. Steps 3 and 4 are repeated 3–5 times with the physician reengaging a new restrictive barrier after each repetition. Following the final repetition, a final stretch is performed further into the restrictive barrier.

6. Retest.

##### Ribs 6–10

Diagnosis: Rib 8 on the left – exhalation somatic dysfunction

Restriction: Rib 8 on the left demonstrates reduced ability to move into the inhalation position

Position: The physician stands on the left side of the supine patient

**Figure 29:**
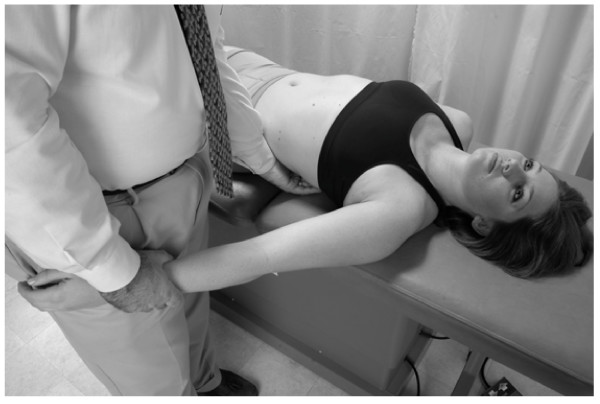
Muscle energy technique for exhalation restriction of left eighth rib.

1. The physician reaches with his/her left hand under the patient to grasp the rib angle of the dysfunctional 8^th ^rib and applies traction in an inferolateral direction.

2. The physician abducts the patient's left arm and places his/her right leg against the patient's distal forearm.

3. The patient inhales while the physician moves the rib inferiorly into the restrictive barrier.

4. The patient holds in his/her breath for 3–5 seconds while contracting the serratus anterior by bringing his/her left arm toward his/her side (adduct) against the unyielding counterforce provided by the physician's right leg (Fig. 29).

5. Steps 3 and 4 are repeated 3–5 times with the physician reengaging a new restrictive barrier after each repetition. Following the final repetition, a final stretch is performed further into the restrictive barrier.

6. Retest.

##### Ribs 11–12

Diagnosis: Rib 12 on the right – exhalation somatic dysfunction

Restriction: Rib 12 on the right demonstrates reduced ability to move into the inhalation position

Position: The physician stands on the left side of the prone patient

1. The physician pulls the patient's legs 15–20° to the left, in this example, away from the dysfunctional rib.

2. The physician places the thenar eminence of his/her left hand just below the inferior border and medial to the angle of the dysfunctional 12^th ^rib.

3. The physician's right hand grasps the right ASIS and pulls the patient's innominate superiorly to engage the restrictive barrier.

4. The patient pulls his/her right hip toward the table for 3–5 seconds against the physician's unyielding counterforce.

5. The patient then relaxes and the counterforce is ceased until the tissues relax in about 2–3 seconds.

6. Steps 4 and 5 are repeated 3–5 times with the physician reengaging a new restrictive barrier with each repetition. Following the final repetition, a final stretch is performed further into the restrictive barrier.

7. Retest.

#### Deep cervical soft tissue procedures

Soft tissue technique is defined as: "A direct technique that usually involves lateral stretching, linear stretching, deep pressure, traction and/or separation of muscle origin and insertion while monitoring tissue response and motion changes by palpation."25

##### Long axis kneading

Diagnosis: Cervical Paravertebral Muscle Tightness/Somatic Dysfunction

Position: The patient is supine. The physician stands at the head of the table.

**Figure 30:**
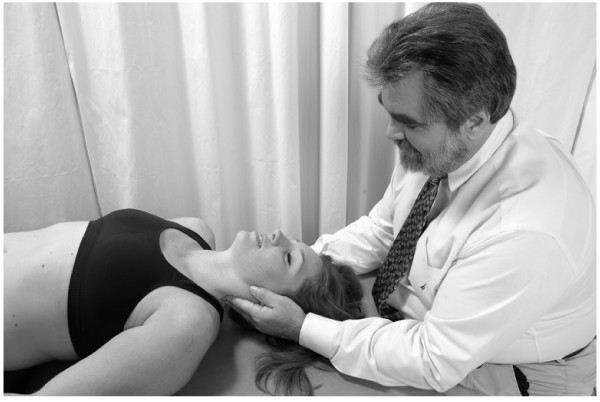
Long axis kneading soft tissue technique.

**Figure 31:**
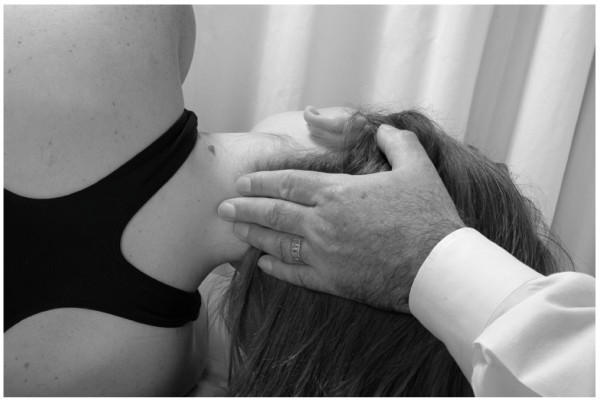
Hand position for long axis kneading soft tissue technique.

1. The physician places his/her finger pads on the cervical paravertebral muscles lateral to the spinous processes (Fig. 30).

2. The physician applies a small amount of anterior pressure, enough to stretch the skin in an inferior direction. The physician then applies deeper pressure as he/she pulls anteriorly, superiorly and laterally (Fig. 31). The physician's fingers do not slide on the skin.

3. When the physician has completed the superior motion, he/she releases the deep pressure.

4. Maintaining enough superficial pressure, he/she takes the skin inferior to the starting point.

5. The physician repeats the above process in an oval, wave-like manner.

6. The physician repeats as many times as necessary to achieve the desired tissue response (as denoted by palpable softening of the tissues, perhaps a sense of increased tissue warmth and/or increased compliancy/flexibility).

7. Retest for appropriate tissue responses.

##### Transverse cervical soft tissue technique

Diagnosis: Left cervical paravertebral muscle tightness/somatic dysfunction

Position: The patient is supine. The physician stands on the right side of the supine patient near the patient's neck.

**Figure 32:**
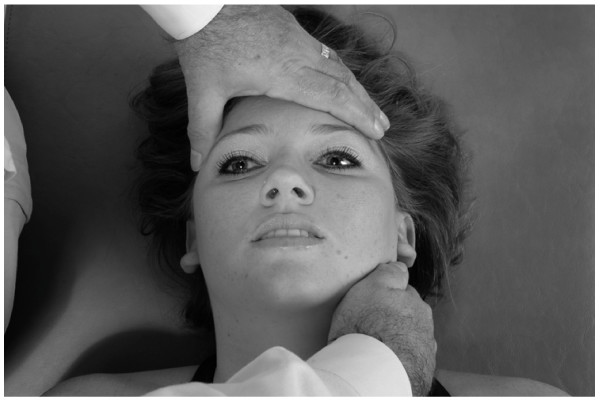
Hand position for transverse cervical soft tissue technique.

**Figure 33:**
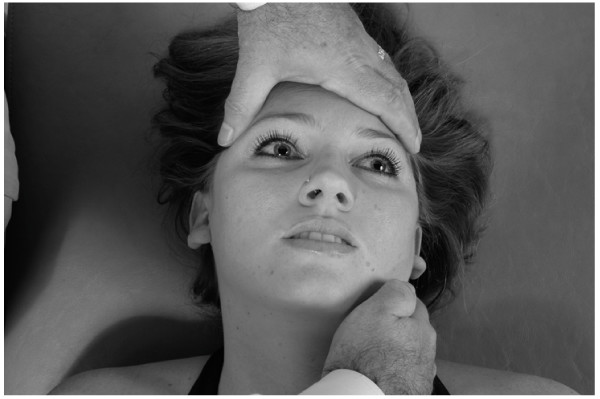
Anterolateral traction on the cervical soft tissues with simultaneous rotation of the patient's head.

1. The physician rests his/her left hand gently on the patient's forehead, while his/her right hand contacts the left side of the neck posteriorly (Fig. 32).

2. The physician's finger pads contact the left paravertebral muscles, with the fingertips just lateral to the spinous processes.

3. The physician gently pushes the forehead away with the left hand, and rotates the head to the left. At the same time the physician draws the left paravertebral muscles laterally and anteriorly until the tissues are at their barrier. The physician resists the cervical rotation slightly with the left hand on the forehead (Fig.33). This creates a dynamic, stretching tension in the muscles and connective tissue.

4. The physician repeats the above process as many tines as necessary to achieve the desired tissue response.

5. The physician treats the right paravertebral muscles in the same manner using the opposite hand placement.

Retest for appropriate tissue responses.

## Competing interests

The author(s) declare that they have no competing interests.

## Authors' contributions

RJH: Conceived the idea for this topic, researched supportive literature, developed the material for the technique section of the manuscript, and wrote the text for the manuscript.

KNH: Assisted with researching supportive literature, wrote a substantial portion of the text for the technique section of the manuscript, assisted with editing of the text, and served as the model for the photographs used in the manuscript.
